# Geologic events coupled with Pleistocene climatic oscillations drove genetic variation of Omei treefrog (*Rhacophorus omeimontis*) in southern China

**DOI:** 10.1186/s12862-015-0572-1

**Published:** 2015-12-21

**Authors:** Jun Li, Mian Zhao, Shichao Wei, Zhenhua Luo, Hua Wu

**Affiliations:** Institute of Evolution and Ecology, International Research Centre of Ecology and Environment, School of Life Sciences, Central China Normal University, 152 Luoyulu, Hongshan District, Wuhan, 430079 China

**Keywords:** Population genetic structure, Demographic history, Tectonic events, Pleistocene glaciations, *Rhacophorus omeimontis*, Southern China

## Abstract

**Background:**

Pleistocene climatic oscillations and historical geological events may both influence current patterns of genetic variation, and the species in southern China that faced unique climatic and topographical events have complex evolutionary histories. However, the relative contributions of climatic oscillations and geographical events to the genetic variation of these species remain undetermined. To investigate patterns of genetic variation and to test the hypotheses about the factors that shaped the distribution of this genetic variation in species of southern China, mitochondrial genes (cytochrome b and NADH dehydrogenase subunit 2) and nine microsatellite loci of the Omei tree frog (*Rhacophorus omeimontis*) were amplified in this study.

**Results:**

The genetic diversity in the populations of *R. omeimontis* was high. The phylogenetic trees reconstructed from the mitochondrial DNA (mtDNA) haplotypes and the Bayesian genetic clustering analysis based on microsatellite data both revealed that all populations were divided into three lineages (SC, HG and YN). The two most recent splitting events among the lineages coincided with recent geological events (including the intense uplift of the Qinghai-Tibet Plateau, QTP and the subsequent movements of the Yun-Gui Plateau, YGP) and the Pleistocene glaciations. Significant expansion signals were not detected in mismatch analyses or neutrality tests. And the effective population size of each lineage was stable during the Pleistocene.

**Conclusions:**

Based on the results of this study, complex geological events (the recent dramatic uplift of the QTP and the subsequent movements of the YGP) and the Pleistocene glaciations were apparent drivers of the rapid divergence of the *R. omeimontis* lineages. Each diverged lineages survived *in situ* with limited gene exchanges, and the stable demographics of lineages indicate that the Pleistocene climatic oscillations were inconsequential for this species. The analysis of genetic variation in populations of *R. omeimontis* contributes to the understanding of the effects of changes in climate and of geographical events on the dynamic development of contemporary patterns of genetic variation in the species of southern China.

**Electronic supplementary material:**

The online version of this article (doi:10.1186/s12862-015-0572-1) contains supplementary material, which is available to authorized users.

## Background

Recently, two primary drivers, namely, Pleistocene climatic oscillations and geological events, have been used to explain the contemporary genetic variation within Northern Hemisphere species. In the Pleistocene, climatic cycles generally caused populations to diverge and produce new lineages, additionally affecting the demographics of the populations. In concrete terms, the species were restricted to refugia during glacial periods and subsequently expanded into new habitats during interglacial periods. As a result, the geographic distributions of populations and the genomes of species were markedly affected [1, 2]. However, genetic differentiation is affected differently by geological events [3, 4]. The formation of geographical barriers, including the uplift of range systems, resulted in limited gene flow between populations, thereby providing opportunities for divergence due to genetic drift and natural selection [5, 6]. The current genetic variation within a species is shaped by these two processes working singly or in combination [2, 7, 8].

The indigenous species of southern China are diverse and have complex evolutionary histories. The unique geographic and climatic features in this area most likely shaped the distributions of genetic variation for these species [4, 7, 9]. First, the terrain of southern China is extremely complex and is characterized by numerous mountains and deep valleys such as the Yun-Gui Plateau (YGP) and the Sichuan Basin. The three-stepped geomorphology of the Chinese mainland, bounded by the Kunlun-Qilian-Hengduan mountain ranges and the Daxinganling-Taihang-Wushan-Xuefeng (the western side of the YGP) mountain ranges, is a salient feature of this region [10, 11]. Topographic features can serve as geographic barriers to prevent gene flow between populations and therefore facilitate genetic divergence [4, 12–14]. Second, three to five intermittent glaciations during the Pleistocene have been recorded in the high mountains of southern China [15–17]. Cycles of glaciations can drive postglacial expansion of populations and therefore affect the patterns of genetic variation [9, 18–20]. Thus, species native to southern China are appropriate models to clarify the contributions of geography and climate to contemporary genetic divergence and diversification.

However, for the species in southern China, few studies have focused on the mechanisms that shaped the current patterns of genetic variation, and the relative importance of these mechanisms remains controversial. In studies of birds such as the great tit (*Parus major*) [20] and the Chinese Hwamei (*Leucodioptron canorum canorum*) [9], lineage diversification and population expansion during the Pleistocene, reveal the large effects of climatic changes on the genetic variation within these species. However, studies of other species have found that geological events were predominant in affecting genetic variation and that the role of Pleistocene climatic changes was relatively weak. For example, studies on cold water fish and frogs show that genetic lineage divergence coincided with tectonic events (e.g., the uplift of the Ailao Mountains and the QTP), and the lineages were persistent and stable or experienced partial secondary contact during the Pleistocene [4, 7, 8]. These discrepancies in the importance of geography and climate can be attributed to two factors. First, the relative contributions of climate and geology to species genetic variation most likely differ because species inhabit different regions in southern China with diverse topographies and microhabitats. For example, the species that live in continuous mountains responded differently to the glacial oscillations from those that live in isolated mountains, with possibly different population demographics within a species [19]. Second, species with different levels of mobility most likely responded differently to historical processes. For example, highly mobile species (e.g., birds and rats) were more likely to escape from inhospitable habitats to refuges during glaciations and then to disperse long distances to new habitats after ice ages; the result was structured genetic diversity patterns and expanded demographics [19, 21]. In contrast, species with low mobility were likely to remain *in situ* and consequently were more sensitive than highly mobile species to topographical changes. Thus, further studies are required for a comprehensive understanding of the influences of Pleistocene climate changes and geological events on genetic variation in the species of southern China.

Amphibians are ideal organisms for detecting the effects of various processes on genetic variations [22]. The Omei tree frog (*Rhacophorus omeimontis*), an arboreal anuran endemic to the subtropical mountain forests of southern China [23], was selected as the object of study because of particular advantages. First, previous research based on fossil records has suggested that Rhacophorid species dispersed from India to Asia at 46 to 57 Ma B.P. [24]. This long-term persistence in Asia allows them to harbor ample genetic legacy of evolutionary processes. Second, unlike the amphibians that spend much of the time in streams or ponds, such as *Q. boulengeri* (dwells in cold montane streams) [7], adult Omei tree frogs typically live on land. Therefore, these terrestrial frogs are suitable to detect the effects of shifts in the Pleistocene climate because they are more likely affected by environmental changes than aquatic species. Third, their low mobility makes them uneasy to disperse through geographical barriers and thus facilitating genetic divergence [25], which means that they are more likely to be affected by geological events.

In this paper, we determined the contributions of geological events and Pleistocene climatic changes to the contemporary genetic patterns of *R. omeimontis* with analyses of mitochondrial DNA (mtDNA) sequences (cytochrome b, cytb and NADH dehydrogenase subunit 2, ND2) and nine microsatellite loci. Specifically, if the geological events are the most important driver, then significant genetic differentiation between lineages is expected. In this case, the lineages diverged concurrently with geological events and remained stable during the Pleistocene. Alternatively, the Pleistocene climatic shifts likely contributed to genetic diversification during the evolution of *R. omeimontis* when the lineage divergence coincided with the Pleistocene glaciations, and/or significant expansion occurred after glacial periods.

## Methods

### Ethics statement

The animal experiments were performed under an animal ethics approval granted by the School of Life Sciences, Central China Normal University. Our sampling procedures did not significantly affect the survival of the studied species.

### Sample collection

**Fig. 1:**
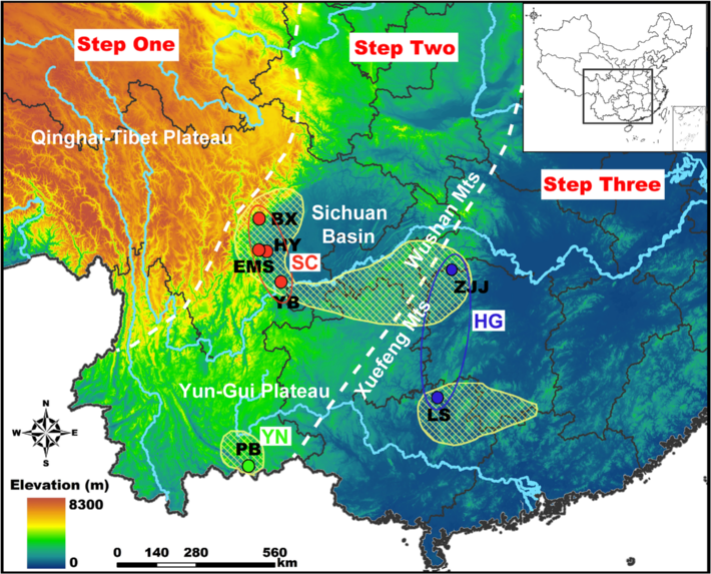
Sampling locations of the populations of *R. omeimontis*. The locality codes and coordinates are presented in Table 1. The three terrain steps in China and the primary mountain systems are marked. The yellow shadow areas represent the distribution range of *R. omeimontis* on the IUCN range map (http://www.iucnredlist.org/). The genetic lineages are labeled red, green and blue for the lineages SC, YN and HG, respectively

From seven localities in the montane forests of southern China, a total of 196 samples were obtained (Fig. 1). The individuals were all used for mtDNA sequencing, and 175 of them were used for microsatellite allele genotyping (Table 1). For each locality, tissues from at least 20 specimens were collected for DNA analyses. All samples were collected with the permission of the local forestry bureaus or the natural reserves. Specifically, for localities in Sichuan Province, sampling was permitted by the EMeiShan (EMS) city and HongYa (HY) country forestry bureaus, the Fengtongzhai National Natural Reserve in BaoXing (BX) city, and the Laojunshan National Natural Reserve in YiBin (YB) city. Sampling in ZhangJiaJie (ZJJ, Hunan Province) was granted by the Badagongshan National Natural Reserve. Sampling in LongSheng (LS, Guangxi Province) and PingBian (PB, Yunnan Province) were permitted by the Huaping National Natural Reserve and the Daweishan National Natural Reserve, respectively. The sampling localities on the second terrain step of China were EMS, HY, BX and YB of Sichuan Province and PB of Yunnan Province. The populations of ZJJ and LS were located on the third terrain step (Fig. 1). Toe tips were clipped from *R. omeimontis* (which is evaluated as least concern on the IUCN red list of threatened species, http://www.iucnredlist.org/) to provide the tissue specimens. An antiseptic was used both on the toes and the scissors to reduce infections. The frogs were released immediately after the surgery and the tissue samples were immersed in 95 % ethyl alcohol.

**Table 1:** Genetic diversity indices of seven populations of *R.omeimontis*

Code	County, province	Coordinates	MtDNA				Microsatellite loci				
			*N*	*N.H*	*h*	*π* (%)	*N*	*N.A.*	*Ho*	*He*	*A* ^*R*^
EMS	Emeishan, Sichuan	29.58 N	22	7	0.81	0.13	23	12.14	0.78	0.80	10.37
		103.28 E									
HY	Hongya, Sichuan	29.58 N	34	11	0.85	0.16	34	11.00	0.73	0.79	9.18
		103.13 E									
BX	Baoxing, Sichuan	30.53 N	30	2	0.50	0.03	18	6.71	0.72	0.69	6.45
		102.93 E									
YB	Yibin, Sichuan	28.07 N	29	7	0.80	0.39	29	11.57	0.79	0.82	9.56
		104.02 E									
ZJJ	Zhangjiajie, Hunan	29.67 N	32	9	0.85	0.18	21	10.29	0.68	0.82	9.18
		110.03 E									
LS	Longsheng, Guangxi	25.06 N	20	3	0.28	0.03	21	9.86	0.71	0.83	8.89
		109.92 E									
PB	Pingbian, Yunnan	22.90 N	29	5	0.32	0.05	29	8.29	0.57	0.72	7.15
		103.68 E									
Total			196	39	0.95	1.91	175	22.71	0.71	0.87	12.70

*N*, number of individuals; *N.H*., number of haplotypes identified from concatenated mtDNA sequences; *h*, haplotype diversity; *π*, nucleotide diversity; *N.A.*, number of microsatellite alleles; *Ho*, observed heterozygosity; *He*, expected heterozygosity; and *A* ^*R*^, allelic richness

### Laboratory protocols

The total DNA was extracted from the toe tips using a TIANamp Genomic DNA kit (TIANGEN Biotech Co. Ltd., Beijing, China) according to the manufacturer's protocol. We designed two pairs of primers to amplify the fragments of mitochondrial cytb (ROcytb1, CCGCAACACGTAAATCTCAC; ROcytb2, GGGGTGAAGTTATCTGGGTC) and ND2 (ROND2L, GGCCCATACCCCAACAAT; ROND2R, GGCTTTGAAGGCCTTTGG). The PCR was conducted in a 30 μl reaction volume that contained 1 μl of genomic DNA, 15 μl of Premix *ExTaq* (TaKaRa, Kusatsu, Japan), 1.2 μl of forward and reverse primers and 11.6 μl of ddH_2_O. All reactions were conducted as follows: an initial denaturation at 94 °C for 3 min, followed by 33 cycles of PCR [94 °C/1 min, 53 °C (cytb) or 55 °C (ND2)/1 min, 72 °C/1 min] and a final extension at 72 °C for 10 min. After a distinct band of the expected fragment size was confirmed, the PCR products were sent to a commercial sequencing company (Nuosai Biotech Inc., Wuhan, China) for sequencing in both directions using an ABI 3730XL DNA Analyzer (Applied Biosystems, Foster, CA, USA). The identical PCR primers were used for the sequencing.

Nine microsatellite loci (OMTF1, OMTF3, OMTF4, OMTF5, OMTF6, OMTF7, OMTF9, OMTF10, and OMTF11) were selected from 11 previously published *R. omeimontis* loci based on the polymorphic evaluations of each locus [26]. The forward primers were all marked with fluorescent dye (FAM, HEX and TAMRA). The PCR amplifications were conducted in a total volume of 10 μl that contained 0.3 μl of genomic DNA, 5 μl of Premix *rTaq* (TaKaRa, Kusatsu, Japan), 0.4 μl of forward and reverse primers and 3.9 μl of ddH_2_O. The reactions began at 95 °C for 5 min, followed by a series of 31 cycles (94 °C/30 s, *Tm*/30 s, 72 °C/45 s) and a final extension of 72 °C for 10 min. The annealing temperature (*Tm*) was set within 53–60 °C (for details, see Additional file 1: Table S1). The positive products were assessed using an ABI 3730XL DNA Analyzer (Applied Biosystems, Foster, CA, USA) and scanned using GeneMapper 4.0 (Applied Biosystems, Foster, CA, USA). The results were read with GeneMarker 1.3 (SoftGenetics, State College, PA, USA) and manually input into Microsoft Excel for format transformation and analyses.

### Data analyses

#### Raw data processing

For the mtDNA data, both directions of the cytb and ND2 sequences were assembled into one contig using SeqMan Promode in LaserGene 7.1.0 (Dnastar, Inc.). The sequences were all aligned using ClustalW in MEGA 5.0 [27]. A partition-homogeneity test was conducted in PAUP* 4.10b [28] with 1000 replicates to determine whether the cytb and ND2 fragments could be concatenated into one dataset [29]. Because the results of the test were not significant (*p* = 0.228), the combined sequences were used in subsequent analyses. For the microsatellite data, the allele values that represented lengths of amplified fragments were transformed into the Arlequin frequency, GenePop2-digit and Fstat formats in Excel using the Microsatellites add-in (MS tools.xla, [30]). Before the analyses, each locus was tested for linkage disequilibrium and deviation from Hardy-Weinberg equilibrium using Genepop (http://genepop.curtin.edu.au/). Significance thresholds were adjusted using the Bonferroni correction. Genotyping errors, including stuttering errors, null alleles and large allele dropouts, were detected using Micro-Checker 2.2.3 [31].

### Genetic diversity and population differentiation

Genetic diversity indices were calculated for both the mtDNA sequences and microsatellite data. For the mtDNA sequences, nucleotide diversity (*π*, [32]) and haplotype diversity (*h*, [33]) were calculated in DNASP 5.0 [34]. For the microsatellite data, molecular diversity indices, including the number of alleles (*N.A.*) and the observed (*Ho*) and expected heterozygosities (*He*), were calculated in Arlequin 3.1 [35]. Allelic richness (*A*^*R*^) which excludes the influences of sample size was calculated in FSTAT 2.9.3.2 [36].

We then evaluated the genetic variation of populations of *R. omeimontis*. First, pairwise *F*-statistics were estimated to evaluate the level of genetic differentiation between population pairs. The *Фst* (for molecular sequence data) and *Rst* (for microsatellite data) are useful *F-statistics* in which they evaluate information not only on the particular haplotypes/alleles frequency but also on the mutational distance [37]. The estimations of pairwise *Фst* (Jukers & Cantor distance matrix) and *Rst* (sum of squared differences, *Rst*-like) values were conducted in Arlequin 3.1 with 10,000 permutations. Then, we analyzed the isolation by distance (IBD) pattern across the seven populations. The Euclidean geographic distances between population pairs were estimated using ArcGis 9.3 [38]. Mantel tests between the geographical distances and the Slatkin linearized *Фst* (*Rst*) matrices were conducted in Arlequin 3.1 with 10,000 permutations. Moreover, we conducted a hierarchical analysis of the molecular variance (AMOVA [39]) in Arlequin 3.1 with 10,000 permutations. The population groups were defined based on the results of phylogenetic analyses. Additionally, to intuitively investigate the genetic divergence among genetic lineages, we calculated pairwise genetic distances for the combined and the cytb sequences based on the Kimura 2-parameter model as implemented in MEGA 5.0 (with 10,000 bootstraps).

### Matrilineal genealogy and genetic structure

According to the large-scale phylogeny of Amphibia and Rhacophoridae [24, 40], three related species, *R. schlegelii, Polypedates megacephalus* and *Buergeria buergeri* (GenBank Accession numbers: AB202078.1, AY458598.1 and AB127977.1, respectively), were selected as the out-groups. These sequences were aligned with our data and combined into an entire dataset. Before reconstruction of the phylogenetic trees, the best-fitting nucleotide substitution model (the general time reversible + proportion of invariable sites + gamma distribution, GTR + I + G) was selected using ModelTest 3.7 [41]. The maximum parsimony (MP) and Bayesian inferences (BI) methods were used to identify the phylogenetic relationships among the mitochondrial haplotypes. The MP tree was constructed in PAUP*4.10b. Heuristic searches were executed for 1000 random addition replicates. The BI tree was constructed in MrBayes 3.1.1 [42]. The Markov chain Monte Carlo (MCMC) analysis was initially performed with four chains at a temperature of 0.2, with the chains run for 5,000,000 cycles. Additionally, the haplotype median-joining network was constructed using the MP method in NETWORK 4.6.1.2 (Fluxus Technology, Suffolk, UK).

The genetic relationships of these populations based on the microsatellite data were deduced using a Bayesian clustering method implemented in STRUCTURE 2.3.1 [43]. The admixture model and the allele frequencies correlated model were used to estimate the number of probable clusters (*K* value). The lengths of the MCMC iterations were set to 100,000 with a burn-in period of 100,000. During the run, the range of *K* values was set to 1 to 12, and each *K* was independently run 20 times. The most probable *K* value was selected according to the peak value of the average log likelihood [Ln P(*X*/*K*)] and the *ΔK* statistic for a given *K* [44].

### Estimation of divergence time between lineages

To estimate the divergence time between lineages, a suitable molecular clock was established using the relative rate tests in PHYLTST [45]. The best-fitting substitution model was assessed in ModelTest 3.7. A range of substitution rates of 0.65–1.00 % per Ma, which included the mtDNA substitution rates of most amphibians [46–49], was used to calculate the divergence time among lineages in BEAST 1.5.4 [50]. Analyses were conducted for 30 million generations using a uniform speciation (Yule) for each prior tree. Each parameter was evaluated in TRACER 1.5 based on the effective sample size (ESS) value. To ensure the accuracy of the results, we combined three independent results using LogCombiner 1.5.4 and constructed the time tree in TreeAnnotator 1.5.4 using a 10 % burn-in period.

### Demographic analyses

We applied three common methods to assess the demographic histories of the three lineages that were inferred from the phylogenetic trees. First, a mismatch distribution was simulated for each lineage to test the hypothesis of recent population growth. A goodness-of-fit test was used to determine the smoothness of the observed mismatch distribution (using Harpending's raggedness index, *Rag*) and the degree of fit between the observed and simulated data (using the sum of squares deviation, *SSD*) [51]. Generally, a smooth and unimodal distribution pattern with non-significant *p-*values for the *SSD* implies a significant expansion signal for a population. Second, the neutrality tests including Tajima's *D* [52] and Fu’s *Fs* [53] were conducted to estimate the demographic expansions of the lineages. In general, significant negative values of Tajima's *D* and Fu's *Fs* imply sudden demographic growth. These two analyses were implemented in Arlequin 3.1 with 10,000 bootstrap replicates. Third, Bayesian skyline plots (BSPs) were generated for all combined sequences of each lineage excluding the out-groups in BEAST 1.5.4, which is a method that reliably reconstructs changes in effective population size across evolutionary time scales from sequence data [54]. The suitable substitution models for the three lineages were selected according to Modeltest 3.7. The analyses were conducted with identical settings to those used for the estimation of divergence time, with the exception that the prior tree was set as the coalescent: Bayesian Skyline.

## Results

### Genetic diversity and population differentiation

For the mtDNA sequencing, a combined mtDNA sequence that included 672 bp cytb and 988 bp ND2 sequences was obtained from each of the 196 individuals. No insertions, deletions or stop codons were found in the entire sequences. Thirty-nine haplotypes were identified and deposited in GenBank (Accession Nos. KP895611-KP895688). For the entire population, the haplotype and nucleotide diversity were relatively high (*h* = 0.95, *π* = 1.91 %). Among the seven populations, the haplotype and nucleotide diversity of the BX, LS and PB populations were lower than those in the other four populations (Table 1). For the microsatellite genotyping, artificial errors such as large allele dropouts and stuttering were not detected. The OMTF5 locus presented null alleles and significant deviation from Hardy-Weinberg equilibrium (HWE) across all populations. Of the nine loci, three pairs (OMTF3 & OMTF4, OMTF4 & OMTF5 and OMTF5 & OMTF1) showed significant linkage disequilibrium. Therefore, to guarantee the reliability of results, we excluded two loci (OMTF4 and OMTF5) in the subsequent analyses. For the entire population, the genetic heterozygosity was relatively high (*Ho* = 0.71, *He* = 0.87, Table 1). Across the seven populations, the number of microsatellite alleles (*N.A.*) ranged between 6.71 and 12.14, and the ranges of the *Ho*, *He* and *A*^*R*^ value were 0.57–0.79, 0.69–0.83 and 6.45–10.37, respectively.

Among the thirty-nine haplotypes identified, only five haplotypes (H3, H4, H5, H7 and H8) were shared by two populations (EMS and HY), with the remaining haplotypes (34 of 39) restricted to one population (see Additional file 2: Table S2). Thus, based on these results, the genetic differentiation among populations was extensive. To further evaluate the genetic differentiation among populations, we conducted pairwise *F-statistics*. The patterns based on the mtDNA and the microsatellite analyses were similar. The genetic differentiation between all population pairs was significantly different from zero except for the four populations in Sichuan Province (EMS, HY, BX and YB), indicating detectable genetic differentiations (Table 2). Additionally, significant correlations between geographical distances and the Slatkin linearized *Фst* (*Rst*) matrices were detected (Table 3), which suggested that the genetic differentiation among populations was correlated with their geographical distributions.

**Table 2:** Pairwise *F-statistics* of seven populations inferred from mtDNA and microsatellite data

	EMS	HY	BX	YB	ZJJ	LS	PB
EMS	0	**0.012**	**0.002**	**0.004**	0.377*	0.598*	0.236*
HY	0.056*	0	**0.016**	**−0.009**	0.455*	0.659*	0.312*
BX	0.719*	0.617*	0	**0.004**	0.482*	0.647*	0.327*
YB	0.191*	0.240*	0.537*	0	0.456*	0.646*	0.332*
ZJJ	0.974*	0.972*	0.982*	0.954*	0	0.524*	0.134*
LS	0.987*	0.982*	0.995*	0.960*	0.563*	0	0.586*
PB	0.984*	0.979*	0.992*	0.957*	0.943*	0.982*	0

Values above the diagonal are the *Rst* values based on the microsatellite data, and values below the diagonal are the *Фst* values based on the mtDNA data. * indicates *p* < 0.05; bold values indicate *p* > 0.05

**Table 3:** Isolation by distance pattern detected at different hierarchical levels

	mtDNA	Microsatellites
Correlation coefficient (*r*)	0.629*	0.615*
Significance (*p*)	0.001	0.016
Determination ratio	0.396	0.378

The determination ratio is the percentage of genetic variations explained by geographical distance. * indicates *p* < 0.05

### Genetic structure

**Fig. 2:**
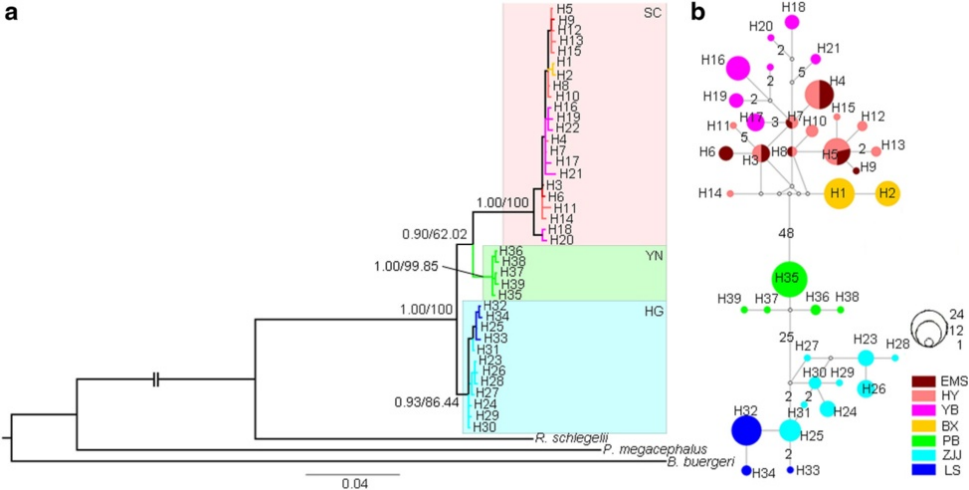
Phylogenetic relationships and haplotype network based on the mtDNA haplotypes of *R. omeimontis*. (**a**) Phylogenetic tree from Bayesian inference analysis with Bayesian posterior probabilities/maximum parsimony bootstrap values near the branches; and (**b**) Median-joining network with node sizes proportional to the frequencies of haplotypes. The numbers of mutations separating the haplotypes are shown on the branches, except for the one-step mutations. Empty nodes indicate undetected haplotypes

With similar tree topology from the MP and BI analyses, the seven populations of *R. omeimontis* were divided into three lineages (the BI tree is shown in Fig. 2a). The haplotypes of the populations ZJJ (Hunan Province) and LS (Guangxi Province), which were distributed in the third terrain step, were clustered into the HG lineage. The haplotypes from the populations in Yunnan and Sichuan provinces in the second terrain step were grouped into a clade with two sub-lineages: one lineage (YN) with the haplotypes of the PB population in Yunnan Province and the other (SC) with the haplotypes from the populations of EMS, HY, BX and YB, which were all in Sichuan Province (Fig. 2a). These three lineages were strongly correlated with the geographic distributions of the populations. The haplotype network also displayed three multiple-mutated clusters, which were similar to those inferred from the phylogenetic trees (Fig. 2b).

**Fig. 3:**
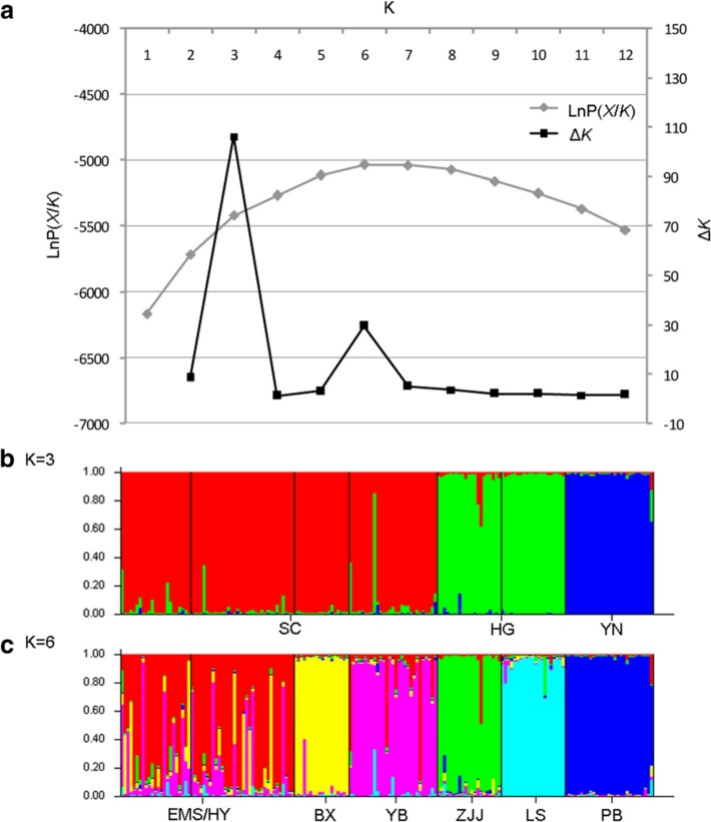
STRUCTURE clustering results deduced from microsatellite alleles within populations of *R. omeimontis*. (**a**) Ln *P*(*X*/*K*) and *ΔK* values as a function of the *K* values according to 20 run outputs; (**b**) STRUCTURE results at *K* = 3, with different colors representing different clusters; and (**c**) STRUCTURE results at *K* = 6, with different colors representing different sub-clusters

The genetic clustering analysis based on the microsatellite data produced a genetic structure that was similar to that of the matriline genealogy and haplotypes network of the mtDNA data. As displayed in Fig. 3, the entire population was divided into three genetic clusters (SC, HG and YN) with a distinct maximum *ΔK* (*ΔK* = 106.22 at *K* = 3, Fig. 3b), although a small peak also occurred at *K* = 6 (*ΔK* = 29.51) (Fig. 3c). These genetic clusters detected by STRUCTURE analysis were consistent with those of the phylogenetic analyses. To intuitively investigate the genetic divergence among the primary lineages, the pairwise average distances were calculated. For the combined sequences, the genetic distances among the primary lineages varied from 1.4 to 2.2 % (Table 4). Because most studies calculate genetic distances using cytb sequences, we also calculated the genetic distances for cytb sequences. The genetic distances of cytb sequences varied from 0.9 to 2.6 % (Table 4). This range is close to the genetic distances in amphibian con-generic species (2–19 %) [55], reflecting a relatively long-term isolation. Furthermore, from the hierarchical AMOVA, the differentiations among lineages made great contributions to the overall genetic variation of these populations (explained 94.67 % of the total variation in mtDNA and 34.90 % in microsatellite data; Additional file 3: Table S3a, b).

**Table 4:** Percentage of genetic distance for combined mtDNA and cytb sequences (Comb/cytb) between primary lineages based on the Kimura 2-parameter model

	SC	HG	YN
SC			
HG	2.2/2.6		
YN	1.5/0.9	1.4/1.7	

### Estimation of divergence time

**Fig. 4:**
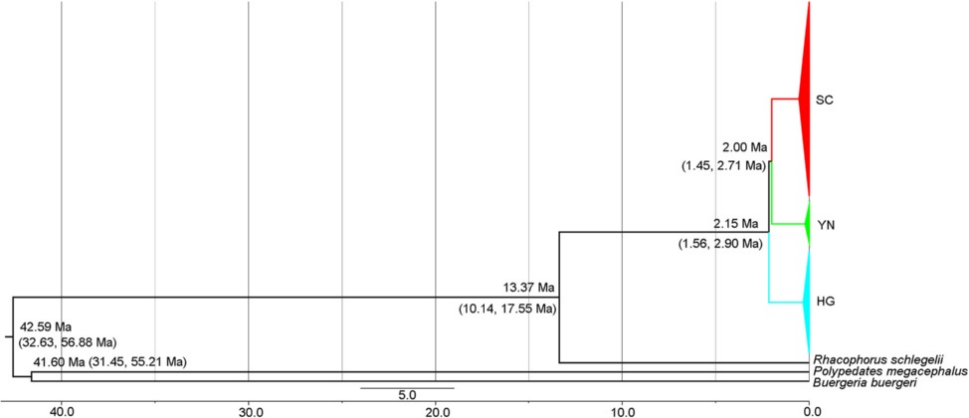
Estimation of divergence time for *R. omeimontis*. The numbers near the primary branches are the estimated split times with 95 % highest posterior density. The three primary lineages are indicated by different colors: red, SC lineage; green, YN lineage; and blue, HG lineage

Because the hypothesis of clock-like evolution was not rejected at the 5 % level in the relative-rate tests, we set a strict molecular clock model for the analyses. As in the phylogenetic tree analyses, the substitution model was set to GTR + I + G. The time since the most recent common ancestor (tmrca) of the entire in-group of *R. omeimontis* was estimated at 13.37 Ma [95 % Highest Posterior Density (HPD), 10.14–17.55 Ma; Fig. 4]. The HG lineage was the first to diverge from the other lineages at approximately 2.15 Ma (95 % HPD, 1.56–2.90 Ma), which was followed by the second divergence between the YN and SC lineages at approximately 2.00 Ma (95 % HPD, 1.45–2.71 Ma). The tmrca for *B. buergeri* and *R. omeimontis* was estimated to be 42.59 Ma (95 % HPD, 32.63–56.88 Ma), which is close to the previous estimation (45.9 Ma, with 95 % HPD, 36.6–57.6 Ma) [56]; the similarity indicates that our results are reliable.

### Demographic history

**Fig. 5:**
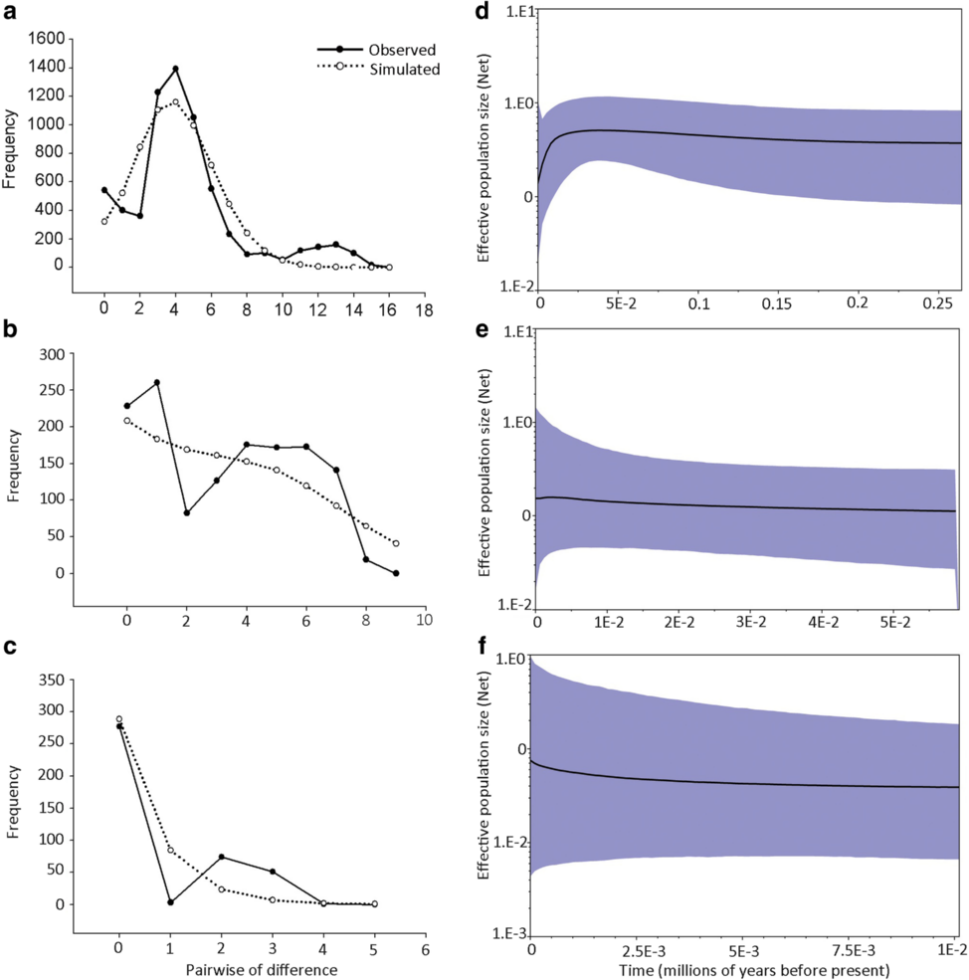
Demographic history analyses for the three lineages according to mismatch distributions and Bayesian skyline plots. (**a**, **b** and **c**) Mismatch distributions for the SC, HG and YN lineages, respectively. The *x* coordinate represents the number of differences in each pair of sequence comparisons, and the *y* coordinate represents the frequencies of pairwise differences. (**d**, **e** and **f**) Bayesian skyline plots for the SC, HG and YN lineages, respectively. The *x*-axis indicates time in Ma BP, and the *y*-axis indicates the effective population size in units of Neτ (the product of effective population size and generation time in Ma). The blue areas represent 95 % highest posterior density

Three methods were used to infer the demographic history of each lineage. For the SC lineage, Tajima's *D* and Fu's *Fs* were negative, but not significant (Table 5), and therefore, the rapid expansion hypothesis was rejected. In the mismatch distribution analysis, although a sudden expansion was not rejected by the goodness-of-fit tests based on non-significant *SSD* and *Rag* values, the sudden expansion hypothesis was not supported because the frequency distribution expressed a multimodal pattern with an initial peak followed by the maximum height and a subsequent small peak (Fig. 5a). Based on the Bayesian skyline plots (BSPs), the effective population size remained stable during the interglacial period (LIG, 0.075–0.125 Ma) [16] and then decreased slightly at approximately 0.012 Ma (early stage of the Holocene) (Fig. 5d). Therefore, signals for significant expansion were not detected in the SC lineage. Similarly, based on the neutrality tests and mismatch distribution analyses (Table 5 and Fig. 5b, c), the rapid expansion hypothesis was rejected for the HG and YN lineages. Additionally, the effective population sizes of these two lineages remained stable over evolutionary timescales as indicated by the BSP curves (Fig. 5e, f).

**Table 5:** Results of the neutrality test and mismatch distribution analysis for the three lineages of *R. omeimontis*

Statistics	Tajima’s *D* (*p*-value)	Fu’s*Fs* (*p*-value)	*Rag* (*p*-value)	*SSD* (*p*-value)
SC lineage	−1.411 (0.078)	−3.430 (0.168)	0.030 (0.172)	0.012 (0.065)
HG lineage	0.737 (0.787)	−1.126 (0.372)	0.028 (0.764)	0.012 (0.612)
YN lineage	−1.112 (0.154)	−1.394 (0.124)	0.504 (0.445)	0.067 (0.081)

*Rag*, Raggedness index; *SSD*, Sum of squares deviations

## Discussion

The Omei tree frog (*R. omeimontis*) is restricted to subtropical forests in southwest China [23]. In this study, based on specimens collected in seven primary areas within the distribution of *R. omeimontis*, we found that the entire population was highly genetically diverse. The high level of mtDNA haplotype diversity for *R. omeimontis* (*h* = 0.95, *π* = 1.91 %) is comparable with that of other amphibians in China, such as *Quasipaa boulengeri* (*h* = 0.96, *π* = 0.97 %) [7] and *Buergeria robusta* (*h* = 0.98, *π* = 1.14 %) [57]. However, the genetic diversity based on microsatellite data was higher in *R. omeimontis* (*Ho* and *He* equal to 0.71 and 0.87, respectively) than that in other amphibians such as the moor frog (*Rana arvalis*, *Ho* = 0.34 and *He* = 0.38) [58] and the wood frog (*Rana sylvatica*, *Ho* = 0.60 and *He* = 0.64 for the highest polymorphic locus) [59]. These high levels of overall genetic diversity were a reflection of the deep divergence among populations.

Pairwise *F-statistics* were calculated to evaluate the genetic differentiation between populations based on the mtDNA sequences and the microsatellite markers. A significant deviation from zero was detected for both *Фst* and *Rst* in all population pairs except for the pairwise *Rst* between populations obtained from Sichuan Province, which indicated detectable genetic differentiation between *R. omeimontis* populations. The incongruence of differentiation pattern between the mtDNA and microsatellite data could be attributed to the different types of inheritance and rates of evolution for the two types of markers and to the unique dispersal pattern of *R. omeimontis*. The polyandry mating system of *R. omeimontis* and the observation that the return rate of *R. omeimontis* females is much higher than males [25, 60] suggest intense female philopatry and historical male dispersal for breeding. Therefore, scarce female gene flow could result in significant mtDNA (maternal inheritance) genetic differentiation among populations, whereas the relatively frequent male dispersal could diminish the genetic differences in microsatellites (parental inheritance). This male-biased gene flow has been reported in many other vertebrates, including birds, lizards and frogs [13].

Based on the broadly consistent results inferred from the mtDNA markers and the microsatellite loci, the seven populations of *R. omeimontis* were divided into three genetic lineages (SC, YN and HG) (Fig. 2) with deep divergences. This genetic structure recovered within *R. omeimontis* was consistent with the complex geological systems in southern China (Fig. 1). Specifically, the SC and YN lineages (located in Sichuan and Yunnan provinces, respectively) and the HG lineage (distributed across Hunan and Guangxi provinces) were separated in the second and third terrain steps of southern China, respectively. Additionally, the SC lineage was restricted to the Sichuan Basin, and the YN lineage was restricted to the YGP (Fig. 1). Moreover, the validity of this geographically structured pattern was supported by the large mutation steps among the haplotype networks (Fig. 2b) and the relatively high genetic distances among the three lineages (Table 4). These results suggest that the genetic lineages diverged deeply and that the divergence might be largely related to specific geologic events within this region. The first splitting event between the HG lineage and the ancestor of the SC and YN lineages was dated to approximately 2.15 Ma with a 95 % HPD of 1.56–2.90 Ma. This splitting event occurred during the periods of recent intense movements of the QTP (1.7–3.6 Ma), which are largely responsible for the formation of the three-stepped terrain in China [11, 61]. The formation of the three-stepped terrain most likely generated geographic barriers that prevented gene flow between the HG lineage and the other populations. Subsequently, dragged by the uplift of the QTP, the YGP also elevated and experienced strong tectonic movements and frequent dry-humid oscillations at 1.4–2.5 Ma [62]. This period of changes for the YGP was consistent with the second splitting event between the SC and YN lineages that occurred at approximately 2.00 Ma (with 95 % HPD, 1.45–2.71 Ma). The tectonic and climatic changes of the YGP might have isolated the YN lineage from the SC lineage and led to the accumulation of autapomorphic mutations. Therefore, the two splitting events within *R. omeimontis* were most likely associated with successive tectonic events (including the recent uplift of the QTP and the subsequent movements of the YGP). Each phase of these movements possibly generated fragmented habitats, which could facilitate local divergence because of limited gene flow among populations for a species with such a low mobility. Similar patterns are also reported in *Leiolepos reevesii* [63], *Leptobrachium ailaonicum* [8], *Rhynchocypris oxycephalus* [4] and *Babina pleuraden* [64]. Hence, the complex geological events were most likely an important factor that drove the lineage divergence of species in southern China.

However, notably, the splitting events within *R. omeimontis* were estimated to have occurred during the Pleistocene (extended from 2.59 Ma to 0.012 Ma [65]) which characterized by cyclic glaciations [1, 4]. The repeated spells of cooling in the Pleistocene could also have increased divergence of the genetic lineages in this environmentally sensitive species. Faced with harsh climates, the species was most likely restricted to several suitable habitats. Combined with the influences of the topographic barriers shaped by geographic events, these populations of *R. omeimontis* were more prone to differentiate rapidly and exhibit deep genetic divergence among lineages; thus, an alternative explanation is provided for the two recent splitting events and the relatively large genetic distances among lineages. Therefore, coupled with the intense tectonic movements, the Pleistocene climatic oscillations were most likely another important factor driving the divergence of the lineages of *R. omeimontis*.

After diverging from one another, the lineages of *R. omeimontis* apparently survived *in situ* to shape the current geographically structured genetic pattern. The gene flow was most likely limited among lineages based on the low genetic similarity and the deep genetic differentiation revealed by the genetic structure analyses. When combined with the low migration capability of this species, the presumption is geographic barriers hindered gene flow and thereby resulted in large genetic differentiation and local evolution. Furthermore, each lineage remained stable during the local evolution. As shown by the demographic history reconstruction, the effective population sizes of all lineages remained constant through evolutionary time. Both the mismatch analyses and neutrality tests did not detect significant signals of rapid expansion, which are the typical responses to climatic oscillations [9, 18–20]. Thus, the Pleistocene climatic oscillations might have had no detectable influences on the demographics of this species. Similarly, for the spiny frog, *Quasipaa boulengeri*, the long-term demographics of lineages during the Pleistocene were also stable in the southern China [7]. Additionally, with distributions in regions identical to those considered in this study, some bird populations remained stable during the Pleistocene, despite population expansion in other regions [19, 66]. Thus, the less significant effects of Pleistocene climatic cycling on demographics might be common to the species in southern China. This phenomenon might be attributed to the buffering effects of topographic features in southern China because the complex mosaic of mountains and valleys provided numerous habitable microhabitats during the Pleistocene ice ages for species to escape from cold climate conditions *in situ* in southern China [9, 67, 68].

## Conclusions

In this paper, we investigated the genetic structure of the populations of *R. omeimontis* and evaluated the relative roles of Pleistocene climatic oscillations and geological events in shaping the current patterns of genetic differentiation of the species in southern China. The levels of genetic diversity were high, and the populations of *R. omeimontis* were clearly differentiated. The phylogenetic reconstructions and the estimation of divergence time indicated that complex geological events (including the recent uplift of the QTP and the subsequent movements of the YGP) coupled with the Pleistocene glaciations drove the genetic divergence of the lineages of *R. omeimontis*. Following the divergence, each lineage was restricted *in situ* and diverged independently without distinct population expansion. Therefore, the Pleistocene climatic oscillations did not appear to influence the demographics of this species, and this phenomenon might be unique for species in general in southern China because of the complex topography. The results of this study will increase our comprehensive understanding of the relative contributions of geographical events and the Pleistocene climatic changes to the current genetic patterns of species in southern China.

## Availability of supporting data

The sequence dataset generated herein is available in the GenBank repository with Accession numbers KP895611-KP895688.

## Additional files

Additional file 1: Table S1. Types of labeled fluorescent dye, annealing temperature (*Tm*) and GenBank accession numbers of microsatellite loci. (DOC 32 kb) Additional file 2: Table S2. Distribution of combined mtDNA sequence haplotypes in seven populations. (DOC 71 kb) Additional file 3: Table S3. a. Hierarchical AMOVA analysis based on mtDNA sequences. b. Hierarchical AMOVA analysis based on microsatellite data. (DOC 33 kb)
