# Supplementary material for: Geologic events coupled with Pleistocene climatic oscillations drove genetic variation of Omei treefrog (Rhacophorus omeimontis) in southern China
Source: BMC Evol Biol. 2015 Dec 21;15:289. doi: 10.1186/s12862-015-0572-1 (PMC4687352; doi:10.1186/s12862-015-0572-1)
Supplement: Additional file 1: Table S1. — Types of labeled fluorescent dye, annealing temperature (Tm) and GenBank accession numbers of microsatellite loci. (DOC 32 kb) [file 12862_2015_572_MOESM1_ESM.doc]

**Additional file 1: Table S1.** Types of labeled fluorescent dye, annealing temperature (*Tm*) and GenBank accession numbers of microsatellite loci.

| Locus | Label type | *Tm* (°C) | GenBank Accession nos. |
| --- | --- | --- | --- |
| OMTF 1 | FAM | 60 | JQ031742 |
| OMTF 6 | TAMRA | 60 | JQ031747 |
| OMTF 9 | HEX | 56 | JQ031750 |
| OMTF 11 | FAM | 56 | JQ031752 |
| OMTF 3 | HEX | 54 | JQ031744 |
| OMTF 4 | TAMRA | 60 | JQ031745 |
| OMTF 5 | FAM | 54 | JQ031746 |
| OMTF 7 | HEX | 53 | JQ031748 |
| OMTF 10 | TAMRA | 60 | JQ031751 |
