# Supplementary material for: Geologic events coupled with Pleistocene climatic oscillations drove genetic variation of Omei treefrog (Rhacophorus omeimontis) in southern China
Source: BMC Evol Biol. 2015 Dec 21;15:289. doi: 10.1186/s12862-015-0572-1 (PMC4687352; doi:10.1186/s12862-015-0572-1)
Supplement: Additional file 2: Table S2. — Distribution of combined mtDNA sequence haplotypes in seven populations. (DOC 71 kb) [file 12862_2015_572_MOESM2_ESM.doc]

**Additional file 2: Table S2.** Distribution of combined mtDNA sequence haplotypes in seven populations.

| Haplotype  name |  | Haplotype distribution | | | | | |
| --- | --- | --- | --- | --- | --- | --- | --- |
|  | EMS  (*N*=7) | HY  (*N*=11) | BX  (*N*=2) | YB  (*N*=7) | ZJJ  (*N*=9) | LS | PB |
|  |  |  |  |  |  | (*N*=3) | (*N*=5) |
| H1 |  |  | 18 |  |  |  |  |
| H2 |  |  | 12 |  |  |  |  |
| **H3** | **3** | **3** |  |  |  |  |  |
| **H4** | **8** | **8** |  |  |  |  |  |
| **H5** | **4** | **10** |  |  |  |  |  |
| H6 | 4 |  |  |  |  |  |  |
| **H7** | **1** | **2** |  |  |  |  |  |
| **H8** | **1** | **1** |  |  |  |  |  |
| H9 | 1 |  |  |  |  |  |  |
| H10 |  | 3 |  |  |  |  |  |
| H11 |  | 1 |  |  |  |  |  |
| H12 |  | 2 |  |  |  |  |  |
| H13 |  | 2 |  |  |  |  |  |
| H14 |  | 1 |  |  |  |  |  |
| H15 |  | 1 |  |  |  |  |  |
| H16 |  |  |  | 11 |  |  |  |
| H17 |  |  |  | 6 |  |  |  |
| H18 |  |  |  | 4 |  |  |  |
| H19 |  |  |  | 4 |  |  |  |
| H20 |  |  |  | 1 |  |  |  |
| H21 |  |  |  | 2 |  |  |  |
| H22 |  |  |  | 1 |  |  |  |
| H23 |  |  |  |  | 5 |  |  |
| H24 |  |  |  |  | 5 |  |  |
| H25 |  |  |  |  | 9 |  |  |
| H26 |  |  |  |  | 6 |  |  |
| H27 |  |  |  |  | 1 |  |  |
| H28 |  |  |  |  | 1 |  |  |
| H29 |  |  |  |  | 1 |  |  |
| H30 |  |  |  |  | 3 |  |  |
| H31 |  |  |  |  | 1 |  |  |
| H32 |  |  |  |  |  | 17 |  |
| H33 |  |  |  |  |  | 1 |  |
| H34 |  |  |  |  |  | 2 |  |
| H35 |  |  |  |  |  |  | 24 |
| H36 |  |  |  |  |  |  | 2 |
| H37 |  |  |  |  |  |  | 1 |
| H38 |  |  |  |  |  |  | 1 |
| H39 |  |  |  |  |  |  | 1 |

*N* = number of haplotypes identified in each population. Haplotypes in bold are shared by populations.
