# Supplementary material for: Geologic events coupled with Pleistocene climatic oscillations drove genetic variation of Omei treefrog (Rhacophorus omeimontis) in southern China
Source: BMC Evol Biol. 2015 Dec 21;15:289. doi: 10.1186/s12862-015-0572-1 (PMC4687352; doi:10.1186/s12862-015-0572-1)
Supplement: Additional file 3: Table S3. — a. Hierarchical AMOVA analysis based on mtDNA sequences. b. Hierarchical AMOVA analysis based on microsatellite data. (DOC 33 kb) [file 12862_2015_572_MOESM3_ESM.doc]

**Additional file 3 Table S3a.** Hierarchical AMOVA analysis based on mtDNA sequences.

| Source of variation | d.f. | Sum of squares | Variance components | Percentage of variation |
| --- | --- | --- | --- | --- |
| Among groups | 2 | 4765.08 | 42.53 | 94.67 |
| Among populations within groups | 4 | 132.39 | 1.16 | 2.57 |
| Within populations | 189 | 234.46 | 1.24 | 2.76 |
| Total | 195 | 5131.93 | 44.92 |  |

**Table S3b.** Hierarchical AMOVA analysis based on microsatellite data.

| Source of variation | d.f. | Sum of squares | Variance components | Percentage of variation |
| --- | --- | --- | --- | --- |
| Among groups | 2 | 15071.31 | 64.92 | 34.90 |
| Among populations within groups | 4 | 4415.87 | 20.00 | 10.75 |
| Among individuals within populations | 168 | 21494.09 | 26.86 | 14.44 |
| Within individuals | 175 | 12988.00 | 74.22 | 39.90 |
| Total | 349 | 53969.28 | 186.00 |  |
